# Supplementary material for: The role of international support programmes in global health security capacity building: A scoping review
Source: PLOS Glob Public Health. 2023 Apr 5;3(4):e0001763. doi: 10.1371/journal.pgph.0001763 (PMC10075474; doi:10.1371/journal.pgph.0001763)
Supplement: S1 Appendix — (DOCX) [file pgph.0001763.s001.docx]

**S1 Appendix: The role of international support programmes in global health security capacity building: an evidence review**

Contents

[A. Literature search strategy, search terms and selection criteria 2](#_Toc127982225)

[B. Preferred Reporting Items for Systematic reviews and Meta-Analyses extension for Scoping Reviews (PRISMA-ScR) Flow Diagram 4](#_Toc127982226)

[C. Preferred Reporting Items for Systematic reviews and Meta-Analyses extension for Scoping Reviews (PRISMA-ScR) Checklist^1,2^ 5](#_Toc127982227)

[D. Search results 8](#_Toc127982228)

[References 25](#_Toc127982229)

## Literature search strategy, search terms and selection criteria

| **Publication types included** | Published peer-reviewed literature (original quantitative & qualitative research articles, systematic reviews, reviews, evidence summaries, viewpoints and commentaries etc.) and grey literature (e.g. relevant published reports from WHO, International Association of National Public Health Institutes (IANPHI) and US Centers for Disease Control and Prevention (US CDC), other published reports and statements) |
| --- | --- |
| **Databases/repositories included** | MEDLINE, Scopus, Global Health, Embase, Emcare, Google Scholar  Institution websites (WHO, US CDC, IANPHI, DFID/FCDO (Department for International Development/Foreign, Commonwealth & Development Office)) |
| **Search limits** | Language: English  Time limit: 2016 to November 2022  Populations: Low and middle-income countries or “Developing Countries” (Medline) |
| **Search/MeSH terms** | 1. **IHR/GHS-related search terms:**   exp International Health Regulations/ *OR*  international health regulat*.tw. *OR*  IHR.tw. *OR*  global health securit*.tw. *OR*  GHS.tw.   1. **Public health systems & strengthening search terms:**   exp "Delivery of Health Care"/ *OR*  (deliver* adj1 health car*).tw. *OR*  health system* strength*.tw. *OR*  exp Public Health Administration/ *OR*  public health admin*.tw. *OR*  national public health institut*.tw. *OR*  aid funded program*.tw.   1. **Emergency preparedness & response/IHR domain terms (general or disease specific):**   exp Disease Outbreaks/ *OR*  disease outbreak*.tw. *OR*  disease surveillance*.tw. *OR*  exp Coronavirus Infections/ *OR*  coronavirus.tw. *OR*  COVID-19.tw. *OR*  exp Hemorrhagic Fever, Ebola/ *OR*  ebola.tw. *OR*  exp Influenza, Human/ *OR*  influenza*.tw. *OR*  swine flu*.tw. *OR*  exp Zika Virus/ *OR*  zika.tw. *OR*  exp Hazardous Substances/ *OR*  hazard* substance*.tw. *OR*  chemical hazard*.tw. *OR*  exp Poisoning/ *OR*  poisoning.tw. *OR*  exp Disaster Planning/ *OR*  disaster plan*.tw. *OR*  emergency prepar*.tw. *OR*  emergency response*.tw. *OR*  exp Epidemiology/ *OR*  epidemiology.tw. *OR*  exp Laboratories/ *OR*  laborator*.tw.  *AND*   1. **LMIC/context specific search terms:**   exp Developing Countries/ *OR*  developing countr*.tw. *OR*  (low and middle income countr*).tw. *OR*  LMIC.tw. |
| **Search Strategies** | **Strategy 1:** 1. *AND* 2. *AND* 3. *AND* 4.  **Strategy 2 (simplified):** 1. *AND* 4.  **Keyword search method:** Combination of subject heading/keyword and free-text terms of databases. Follow-up search of organisation websites for additional results.  Additional backward and forward citation searches performed on relevant publications, e.g. “Lessons learnt from implementation of the International Health Regulations: a systematic review”; A.B. Suthar et al.; *Bulletin of the World Health Organization (Feb 2018); 96:2, p110–121*  Searches performed on:  12/06/2020 (publication dates: January 2016 – June 2020)  19/08/2020 (publication dates: June – August 2020)  21/06/2021 (publication dates: August 2020 – June 2021)  07/11/2022 (publication dates: June 2021 – November 2022) |

1. **Preferred Reporting Items for Systematic reviews and Meta-Analyses extension for Scoping Reviews (PRISMA-ScR) Flow Diagram**


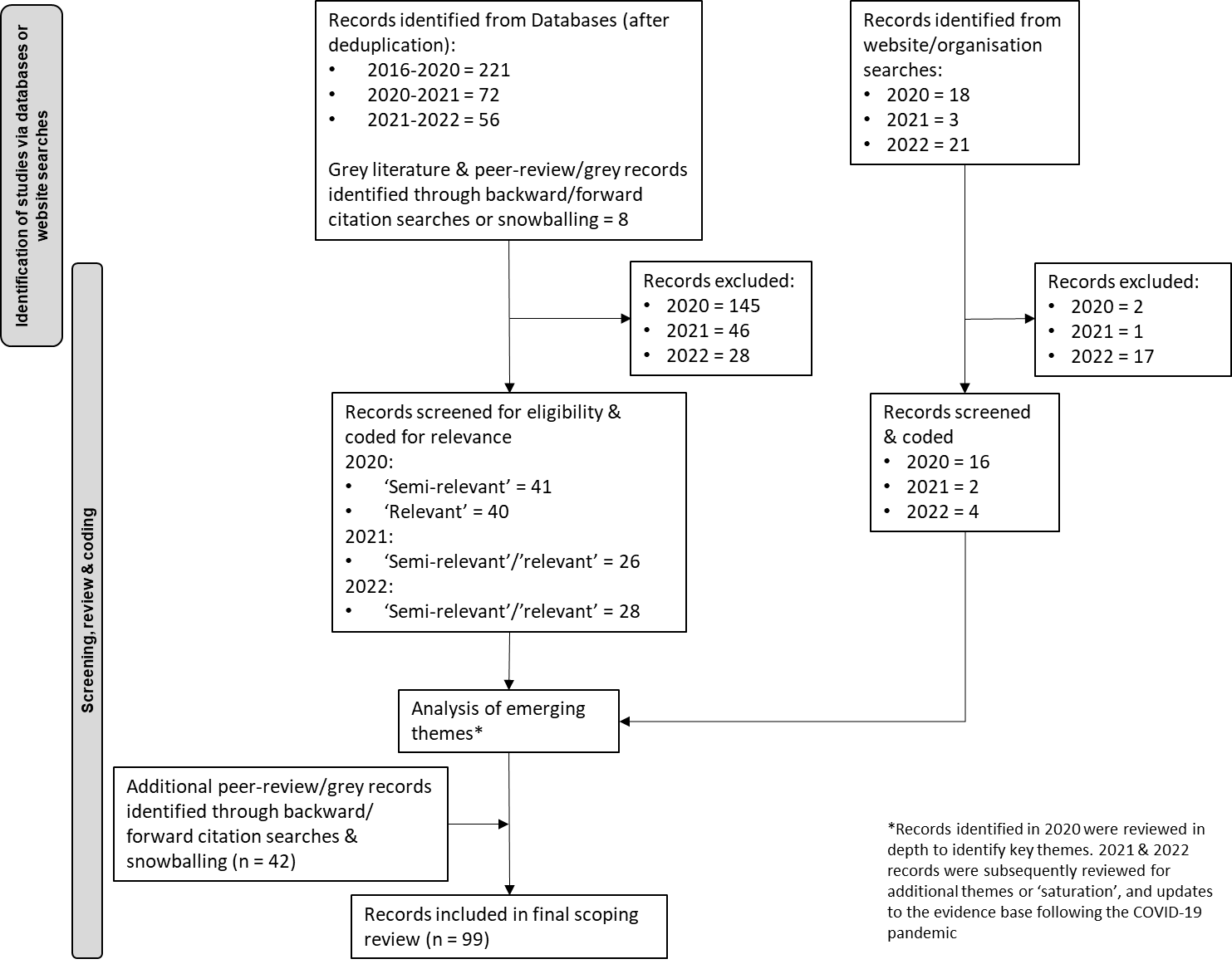


1. **Preferred Reporting Items for Systematic reviews and Meta-Analyses extension for Scoping Reviews (PRISMA-ScR) Checklist^1,2^**

| **SECTION** | **ITEM** | **PRISMA-ScR CHECKLIST ITEM** | **REPORTED ON PAGE #** |
| --- | --- | --- | --- |
| **TITLE** | | | |
| Title | 1 | Identify the report as a scoping review. | 1 (Title) & 5 (“Search strategy and selection criteria”) |
| **ABSTRACT** | | | |
| Structured summary | 2 | Provide a structured summary that includes (as applicable): background, objectives, eligibility criteria, sources of evidence, charting methods, results, and conclusions that relate to the review questions and objectives. | 2 (“Abstract”) |
| **INTRODUCTION** | | | |
| Rationale | 3 | Describe the rationale for the review in the context of what is already known. Explain why the review questions/objectives lend themselves to a scoping review approach. | 3-5 (“Introduction”) |
| Objectives | 4 | Provide an explicit statement of the questions and objectives being addressed with reference to their key elements (e.g., population or participants, concepts, and context) or other relevant key elements used to conceptualize the review questions and/or objectives. | 5 (“Search strategy and selection criteria”) |
| **METHODS** | | | |
| Protocol and registration | 5 | Indicate whether a review protocol exists; state if and where it can be accessed (e.g., a Web address); and if available, provide registration information, including the registration number. | N/A |
| Eligibility criteria | 6 | Specify characteristics of the sources of evidence used as eligibility criteria (e.g., years considered, language, and publication status), and provide a rationale. | 5-6 (“Search strategy and selection criteria”) & S1 Appendix (A) |
| Information sources* | 7 | Describe all information sources in the search (e.g., databases with dates of coverage and contact with authors to identify additional sources), as well as the date the most recent search was executed. | 5-6 (“Search strategy and selection criteria”) & S1 Appendix (A) |
| Search | 8 | Present the full electronic search strategy for at least 1 database, including any limits used, such that it could be repeated. | S1 Appendix (A & D) |
| Selection of sources of evidence† | 9 | State the process for selecting sources of evidence (i.e., screening and eligibility) included in the scoping review. | 5-6 (“Search strategy and selection criteria”) & S1 Appendix (A) |
| Data charting process‡ | 10 | Describe the methods of charting data from the included sources of evidence (e.g., calibrated forms or forms that have been tested by the team before their use, and whether data charting was done independently or in duplicate) and any processes for obtaining and confirming data from investigators. | 5-6 (“Search strategy and selection criteria”) & S1 Appendix |
| Data items | 11 | List and define all variables for which data were sought and any assumptions and simplifications made. | N/A |
| Critical appraisal of individual sources of evidence§ | 12 | If done, provide a rationale for conducting a critical appraisal of included sources of evidence; describe the methods used and how this information was used in any data synthesis (if appropriate). | N/A |
| Synthesis of results | 13 | Describe the methods of handling and summarizing the data that were charted. | 5-6 (“Search strategy and selection criteria”) & S1 Appendix |
| **RESULTS** | | | |
| Selection of sources of evidence | 14 | Give numbers of sources of evidence screened, assessed for eligibility, and included in the review, with reasons for exclusions at each stage, ideally using a flow diagram. | 4-5 (“Search strategy and selection criteria”) & S1 Appendix |
| Characteristics of sources of evidence | 15 | For each source of evidence, present characteristics for which data were charted and provide the citations. | 5-22 |
| Critical appraisal within sources of evidence | 16 | If done, present data on critical appraisal of included sources of evidence (see item 12). | N/A |
| Results of individual sources of evidence | 17 | For each included source of evidence, present the relevant data that were charted that relate to the review questions and objectives. | 5-22 |
| Synthesis of results | 18 | Summarize and/or present the charting results as they relate to the review questions and objectives. | 5-22, Figure 1, Table 1 |
| **DISCUSSION** | | | |
| Summary of evidence | 19 | Summarize the main results (including an overview of concepts, themes, and types of evidence available), link to the review questions and objectives, and consider the relevance to key groups. | 23-26 (“Conclusion”), Figure 1, Table 1 |
| Limitations | 20 | Discuss the limitations of the scoping review process. | 26 (“Conclusion”) |
| Conclusions | 21 | Provide a general interpretation of the results with respect to the review questions and objectives, as well as potential implications and/or next steps. | 23-26 (“Conclusion”), Figure 1, Table 1 |
| **FUNDING** | | | |
| Funding | 22 | Describe sources of funding for the included sources of evidence, as well as sources of funding for the scoping review. Describe the role of the funders of the scoping review. | Financial Disclosure Statement |

## Search results

**12/06/2020 search:**

Embase:

Database(s): **Embase** 1996 to 2020 Week 23

Search Strategy:

| **#** | **Searches** | **Results** |
| --- | --- | --- |
| 1 | exp International Health Regulations/ | 103 |
| 2 | international health regulat*.tw. | 497 |
| 3 | IHR.tw. | 810 |
| 4 | global health securit*.tw. | 421 |
| 5 | GHS.tw. | 3062 |
| 6 | exp "Delivery of Health Care"/ | 2950571 |
| 7 | (deliver* adj1 health car*).tw. | 9619 |
| 8 | health system* strength*.tw. | 925 |
| 9 | exp Public Health Administration/ | 49306 |
| 10 | public health admin*.tw. | 212 |
| 11 | national public health institut*.tw. | 126 |
| 12 | aid funded program*.tw. | 1 |
| 13 | exp Disease Outbreaks/ | 94237 |
| 14 | disease outbreak*.tw. | 5999 |
| 15 | disease surveillance*.tw. | 5374 |
| 16 | exp Coronavirus Infections/ | 17338 |
| 17 | coronavirus.tw. | 20340 |
| 18 | COVID-19.tw. | 14741 |
| 19 | exp Hemorrhagic Fever, Ebola/ | 5851 |
| 20 | ebola.tw. | 9333 |
| 21 | exp Influenza, Human/ | 79019 |
| 22 | influenza*.tw. | 103755 |
| 23 | swine flu*.tw. | 1200 |
| 24 | exp Zika Virus/ | 5546 |
| 25 | zika.tw. | 9073 |
| 26 | exp Hazardous Substances/ | 5458 |
| 27 | hazard* substance*.tw. | 1759 |
| 28 | chemical hazard*.tw. | 766 |
| 29 | exp Poisoning/ | 190486 |
| 30 | poisoning.tw. | 39852 |
| 31 | exp Disaster Planning/ | 10818 |
| 32 | disaster plan*.tw. | 1030 |
| 33 | emergency prepar*.tw. | 1883 |
| 34 | emergency response*.tw. | 3340 |
| 35 | exp Epidemiology/ | 3072372 |
| 36 | epidemiology.tw. | 161992 |
| 37 | exp Laboratories/ | 152094 |
| 38 | laborator*.tw. | 677564 |
| 39 | exp Developing Countries/ | 62326 |
| 40 | developing countr*.tw. | 67502 |
| 41 | (low and middle income countr*).tw. | 18768 |
| 42 | LMIC.tw. | 2463 |
| 43 | 1 or 2 or 3 or 4 or 5 | 4573 |
| 44 | 6 or 7 or 8 or 9 or 10 or 11 or 12 | 2988516 |
| 45 | 13 or 14 or 15 or 16 or 17 or 18 or 19 or 20 or 21 or 22 or 23 or 24 or 25 or 26 or 27 or 28 or 29 or 30 or 31 or 32 or 33 or 34 or 35 or 36 or 37 or 38 | 4054561 |
| 46 | 39 or 40 or 41 or 42 | 114960 |
| 47 | 43 and 44 and 45 and 46 | 12 |
| 48 | limit 47 to (english language and humans and yr="2016 - 2020") | 4 |
| 49 | exp International Health Regulations/ | 103 |
| 50 | international health regulat*.tw. | 497 |
| 51 | IHR.tw. | 810 |
| 52 | global health securit*.tw. | 421 |
| 53 | GHS.tw. | 3062 |
| 54 | exp Developing Countries/ | 62326 |
| 55 | developing countr*.tw. | 67502 |
| 56 | (low and middle income countr*).tw. | 18768 |
| 57 | LMIC.tw. | 2463 |
| 58 | 49 or 50 or 51 or 52 or 53 | 4573 |
| 59 | 54 or 55 or 56 or 57 | 114960 |
| 60 | 58 and 59 | 69 |
| 61 | limit 60 to (english language and humans and yr="2016 -Current") | 22 |

Database(s): **Ovid Emcare** 1995 to 2020 week 23

Search Strategy:

| **#** | **Searches** | **Results** |
| --- | --- | --- |
| 1 | exp International Health Regulations/ | 66 |
| 2 | international health regulat*.tw. | 241 |
| 3 | IHR.tw. | 225 |
| 4 | global health securit*.tw. | 216 |
| 5 | GHS.tw. | 405 |
| 6 | exp "Delivery of Health Care"/ | 1064766 |
| 7 | (deliver* adj1 health car*).tw. | 5714 |
| 8 | health system* strength*.tw. | 513 |
| 9 | exp Public Health Administration/ | 27412 |
| 10 | public health admin*.tw. | 109 |
| 11 | national public health institut*.tw. | 52 |
| 12 | aid funded program*.tw. | 0 |
| 13 | exp Disease Outbreaks/ | 30829 |
| 14 | disease outbreak*.tw. | 1734 |
| 15 | disease surveillance*.tw. | 1680 |
| 16 | exp Coronavirus Infections/ | 4030 |
| 17 | coronavirus.tw. | 2104 |
| 18 | COVID-19.tw. | 2583 |
| 19 | exp Hemorrhagic Fever, Ebola/ | 2666 |
| 20 | ebola.tw. | 3328 |
| 21 | exp Influenza, Human/ | 22867 |
| 22 | influenza*.tw. | 22149 |
| 23 | swine flu*.tw. | 382 |
| 24 | exp Zika Virus/ | 1473 |
| 25 | zika.tw. | 2475 |
| 26 | exp Hazardous Substances/ | 1676 |
| 27 | hazard* substance*.tw. | 430 |
| 28 | chemical hazard*.tw. | 189 |
| 29 | exp Poisoning/ | 55700 |
| 30 | poisoning.tw. | 9212 |
| 31 | exp Disaster Planning/ | 3516 |
| 32 | disaster plan*.tw. | 691 |
| 33 | emergency prepar*.tw. | 1202 |
| 34 | emergency response*.tw. | 1707 |
| 35 | exp Epidemiology/ | 934572 |
| 36 | epidemiology.tw. | 45688 |
| 37 | exp Laboratories/ | 51394 |
| 38 | laborator*.tw. | 129872 |
| 39 | exp Developing Countries/ | 21658 |
| 40 | developing countr*.tw. | 20853 |
| 41 | (low and middle income countr*).tw. | 9416 |
| 42 | LMIC.tw. | 999 |
| 43 | 1 or 2 or 3 or 4 or 5 | 982 |
| 44 | 6 or 7 or 8 or 9 or 10 or 11 or 12 | 1084308 |
| 45 | 13 or 14 or 15 or 16 or 17 or 18 or 19 or 20 or 21 or 22 or 23 or 24 or 25 or 26 or 27 or 28 or 29 or 30 or 31 or 32 or 33 or 34 or 35 or 36 or 37 or 38 | 1147978 |
| 46 | 39 or 40 or 41 or 42 | 38394 |
| 47 | 43 and 44 and 45 and 46 | 3 |
| 48 | limit 47 to (english language and humans and yr="2016 - 2020") | 0 |
| 49 | exp International Health Regulations/ | 66 |
| 50 | international health regulat*.tw. | 241 |
| 51 | IHR.tw. | 225 |
| 52 | global health securit*.tw. | 216 |
| 53 | GHS.tw. | 405 |
| 54 | exp Developing Countries/ | 21658 |
| 55 | developing countr*.tw. | 20853 |
| 56 | (low and middle income countr*).tw. | 9416 |
| 57 | LMIC.tw. | 999 |
| 58 | 49 or 50 or 51 or 52 or 53 | 982 |
| 59 | 54 or 55 or 56 or 57 | 38394 |
| 60 | 58 and 59 | 29 |
| 61 | limit 60 to (english language and humans and yr="2016 -Current") | 8 |

Global Health:

Database(s): Global Health 1910 to 2020 Week 22

Search Strategy:

| **#** | **Searches** | **Results** |
| --- | --- | --- |
| 1 | international health regulat*.tw. | 391 |
| 2 | IHR.tw. | 375 |
| 3 | global health securit*.tw. | 216 |
| 4 | GHS.tw. | 430 |
| 5 | exp Developing Countries/ | 986216 |
| 6 | developing countr*.tw. | 993738 |
| 7 | (low and middle income countr*).tw. | 8597 |
| 8 | LMIC.tw. | 772 |
| 9 | 5 or 6 or 7 or 8 | 996530 |
| 10 | 1 or 2 or 3 or 4 | 1215 |
| 11 | 9 and 10 | 308 |
| 12 | limit 11 to (english language and yr="2016 - 2020") | 138 |

 Global Health:

Database(s): **Global Health** 1973 to 2020 Week 22

Search Strategy:

| **#** | **Searches** | **Results** |
| --- | --- | --- |
| 1 | IHR.tw. | 254 |
| 2 | global health securit*.tw. | 216 |
| 3 | GHS.tw. | 429 |
| 4 | health system* strength*.tw. | 617 |
| 5 | national public health institut*.tw. | 70 |
| 6 | disease outbreak*.tw. | 3742 |
| 7 | disease surveillance*.tw. | 21742 |
| 8 | exp Ebola Haemorrhagic Fever/ | 602 |
| 9 | ebola.tw. | 4682 |
| 10 | influenza*.tw. | 44733 |
| 11 | swine flu*.tw. | 1139 |
| 12 | exp Zika Virus/ | 3635 |
| 13 | zika.tw. | 4483 |
| 14 | hazard* substance*.tw. | 591 |
| 15 | chemical hazard*.tw. | 466 |
| 16 | exp Poisoning/ | 35236 |
| 17 | poisoning.tw. | 34713 |
| 18 | disaster plan*.tw. | 400 |
| 19 | emergency prepar*.tw. | 772 |
| 20 | emergency response*.tw. | 1097 |
| 21 | exp Epidemiology/ | 313438 |
| 22 | epidemiology.tw. | 330060 |
| 23 | exp Laboratories/ | 3322 |
| 24 | laborator*.tw. | 246818 |
| 25 | exp Developing Countries/ | 860746 |
| 26 | developing countr*.tw. | 868267 |
| 27 | (low and middle income countr*).tw. | 8597 |
| 28 | LMIC.tw. | 772 |
| 29 | 1 or 2 or 3 or 4 or 5 | 1551 |
| 30 | 6 or 7 or 8 or 9 or 10 or 11 or 12 or 13 or 14 or 15 or 16 or 17 or 18 or 19 or 20 or 21 or 22 or 23 or 24 | 632693 |
| 31 | 25 or 26 or 27 or 28 | 871051 |
| 32 | 29 and 30 and 31 | 189 |
| 33 | limit 32 to (english language and yr="2016 - 2020") | 106 |

Google Scholar:

“International Health Regulations” (phrase search)

AND

“Developing countries”

2016-2020

Medline:

Database(s): **Ovid MEDLINE and Epub Ahead of Print, In-Process & Other Non-Indexed Citations, Daily and Versions** 1946 to June 05, 2020

Search Strategy:

| **#** | **Searches** | **Results** |
| --- | --- | --- |
| 1 | exp International Health Regulations/ | 27 |
| 2 | international health regulat*.tw. | 483 |
| 3 | IHR.tw. | 789 |
| 4 | global health securit*.tw. | 418 |
| 5 | GHS.tw. | 2355 |
| 6 | exp "Delivery of Health Care"/ | 1070199 |
| 7 | (deliver* adj1 health car*).tw. | 10787 |
| 8 | health system* strength*.tw. | 816 |
| 9 | exp Public Health Administration/ | 15374 |
| 10 | public health admin*.tw. | 587 |
| 11 | national public health institut*.tw. | 94 |
| 12 | aid funded program*.tw. | 1 |
| 13 | exp Disease Outbreaks/ | 98973 |
| 14 | disease outbreak*.tw. | 6226 |
| 15 | disease surveillance*.tw. | 4895 |
| 16 | exp Coronavirus Infections/ | 14522 |
| 17 | coronavirus.tw. | 17279 |
| 18 | COVID-19.tw. | 17195 |
| 19 | exp Hemorrhagic Fever, Ebola/ | 5375 |
| 20 | ebola.tw. | 8301 |
| 21 | exp Influenza, Human/ | 48785 |
| 22 | influenza*.tw. | 114286 |
| 23 | swine flu*.tw. | 930 |
| 24 | exp Zika Virus/ | 3600 |
| 25 | zika.tw. | 7509 |
| 26 | exp Hazardous Substances/ | 14155 |
| 27 | hazard* substance*.tw. | 1395 |
| 28 | chemical hazard*.tw. | 751 |
| 29 | exp Poisoning/ | 156865 |
| 30 | poisoning.tw. | 64168 |
| 31 | exp Disaster Planning/ | 14144 |
| 32 | disaster plan*.tw. | 1260 |
| 33 | emergency prepar*.tw. | 1675 |
| 34 | emergency response*.tw. | 2792 |
| 35 | exp Epidemiology/ | 26696 |
| 36 | epidemiology.tw. | 148537 |
| 37 | exp Laboratories/ | 27930 |
| 38 | laborator*.tw. | 567442 |
| 39 | exp Developing Countries/ | 74496 |
| 40 | developing countr*.tw. | 59033 |
| 41 | (low and middle income countr*).tw. | 16240 |
| 42 | LMIC.tw. | 1740 |
| 43 | 1 or 2 or 3 or 4 or 5 | 3790 |
| 44 | 6 or 7 or 8 or 9 or 10 or 11 or 12 | 1087810 |
| 45 | 13 or 14 or 15 or 16 or 17 or 18 or 19 or 20 or 21 or 22 or 23 or 24 or 25 or 26 or 27 or 28 or 29 or 30 or 31 or 32 or 33 or 34 or 35 or 36 or 37 or 38 | 1165745 |
| 46 | 39 or 40 or 41 or 42 | 126589 |
| 47 | 43 and 44 and 45 and 46 | 5 |
| 48 | limit 47 to (english language and humans and yr="2016 - 2020") | 2 |
| 49 | exp International Health Regulations/ | 27 |
| 50 | international health regulat*.tw. | 483 |
| 51 | IHR.tw. | 789 |
| 52 | global health securit*.tw. | 418 |
| 53 | GHS.tw. | 2355 |
| 54 | exp Developing Countries/ | 74496 |
| 55 | developing countr*.tw. | 59033 |
| 56 | (low and middle income countr*).tw. | 16240 |
| 57 | LMIC.tw. | 1740 |
| 58 | 49 or 50 or 51 or 52 or 53 | 3790 |
| 59 | 54 or 55 or 56 or 57 | 126589 |
| 60 | 58 and 59 | 70 |
| 61 | limit 60 to (english language and humans and yr="2016 -Current") | 18 |

Scopus:

Backward and forward citation search on the reference paper supplied by the requesters:

<https://www.who.int/bulletin/volumes/96/2/16-189100.pdf>

**19/08/2020 search:**

Embase:

Database(s): **Embase** 1996 to 2020 Week 32

Search Strategy:

| **#** | **Searches** | **Results** |
| --- | --- | --- |
| 1 | exp International Health Regulations/ | 108 |
| 2 | (international health regulat* or IHR or global health securit* or GHS).tw. | 4564 |
| 3 | 1 or 2 | 4626 |
| 4 | exp Developing Countries/ | 62856 |
| 5 | (((developing countr* or low) and middle income countr*) or LMIC).tw. | 20015 |
| 6 | 4 or 5 | 80139 |
| 7 | 3 and 6 | 58 |
| 8 | limit 7 to (english language and yr="2020 - 2021") | 4 - 1 after dedup and removing results from previous search |

Database(s): **Ovid Emcare** 1995 to 2020 Week 32

Search Strategy:

| **#** | **Searches** | **Results** |
| --- | --- | --- |
| 1 | exp International Health Regulations/ | 77 |
| 2 | (international health regulat* or IHR or global health securit* or GHS).tw. | 976 |
| 3 | 1 or 2 | 1014 |
| 4 | exp Developing Countries/ | 21818 |
| 5 | (((developing countr* or low) and middle income countr*) or LMIC).tw. | 9862 |
| 6 | 4 or 5 | 30821 |
| 7 | 3 and 6 | 23 |
| 8 | limit 7 to (english language and yr="2020 - 2021") | 3 - 0 after dedup and removing results from previous search |

Global Health:

| **#** | **Query** | **Limiters/Expanders** | **Last Run Via** | **Results** |
| --- | --- | --- | --- | --- |
| S3 | S1 AND S2 | Expanders - Apply equivalent subjects  Search modes - Boolean/Phrase | Interface - EBSCOhost Research Databases  Search Screen - Advanced Search  Database - Global Health | 32 - 29 after dedup and removing results from previous search |
| S2 | SU Developing countries OR TX developing countr* OR TX ( low and middle income countr* ) OR TX LMIC | Limiters - Publication Year: 2020-2021  Expanders - Apply equivalent subjects  Search modes - Boolean/Phrase | Interface - EBSCOhost Research Databases  Search Screen - Advanced Search  Database - Global Health | 20,446 |
| S1 | TX international health regulat* OR TX IHR OR TX Global health securit* OR TX GHS | Limiters - Publication Year: 2020-2021  Expanders - Apply equivalent subjects  Search modes - Boolean/Phrase | Interface - EBSCOhost Research Databases  Search Screen - Advanced Search  Database - Global Health | 53 |

Google Scholar:

International Health Regulations

Limit to: 2020 (results from previous search excluded manually)

No of results: 6

Medline:

Database(s): **Ovid MEDLINE and Epub Ahead of Print, In-Process & Other Non-Indexed Citations and Daily** 1946 to August 13, 2020

Search Strategy:

| **#** | **Searches** | **Results** |
| --- | --- | --- |
| 1 | exp International Health Regulations/ | 37 |
| 2 | (international health regulat* or IHR or global health securit* or GHS).tw. | 3843 |
| 3 | 1 or 2 | 3858 |
| 4 | exp Developing Countries/ | 74882 |
| 5 | (((developing countr* or low) and middle income countr*) or LMIC).tw. | 17225 |
| 6 | 4 or 5 | 87300 |
| 7 | 3 and 6 | 52 |
| 8 | limit 7 to (english language and yr="2020 - 2021") | 6 - 4 after dedup and removing results from previous search |

**21/06/2021 search:**

Embase:

Database(s): Embase 1974 to 2021 June 16

Search Strategy:

| **#** | **Searches** | **Results** |
| --- | --- | --- |
| 1 | exp international health regulation/ | 162 |
| 2 | ("international health regulat*" or IHR or "global health securit*" or GHS).tw. | 5204 |
| 3 | 1 or 2 | 5297 |
| 4 | exp developing country/ | 96294 |
| 5 | ((("developing countr*" or low) and middle income countr*) or LMIC).tw. | 24396 |
| 6 | 4 or 5 | 117586 |
| 7 | 3 and 6 | 68 |
| 8 | limit 7 to (human and english language) | 55 |
| 9 | limit 8 to dd=20200801-20211231 | 3 |
| 10 | limit 8 to rd=20200801-20211231 | 6 |
| 11 | 9 or 10 | 9 |

Emcare:

Database(s): Ovid Emcare 1995 to 2021 Week 23

Search Strategy:

| **#** | **Searches** | **Results** |
| --- | --- | --- |
| 1 | exp international health regulation/ | 115 |
| 2 | ("international health regulat*" or IHR or "global health securit*" or GHS).tw. | 1090 |
| 3 | 1 or 2 | 1142 |
| 4 | exp developing country/ | 22484 |
| 5 | ((("developing countr*" or low) and middle income countr*) or LMIC).tw. | 11779 |
| 6 | 4 or 5 | 33370 |
| 7 | 3 and 6 | 27 |
| 8 | limit 7 to (human and english language) | 21 |
| 9 | limit 8 to dd=20200801-20211231 | 4 |

Global Health:

|  | **Query** | **Limiters/Expanders** | **Last Run Via** | **Results** |
| --- | --- | --- | --- | --- |
| S3 | S1 AND S2 | Expanders - Apply equivalent subjects  Search modes - Boolean/Phrase | Interface - EBSCOhost Research Databases  Search Screen - Advanced Search  Database - Global Health | 19 |
| S2 | SU Developing Countries OR TX "developing countr*" OR TX ( "low and middle income" ) OR TX LMIC | Limiters - Publication Year: 20200801-20211231  Expanders - Apply equivalent subjects  Search modes - Boolean/Phrase | Interface - EBSCOhost Research Databases  Search Screen - Advanced Search  Database - Global Health | 17,864 |
| S1 | TX "international health regulat*" OR TX IHR OR TX "Global health securit*" OR TX GHS | Limiters - Publication Year: 20200801-20211231  Expanders - Apply equivalent subjects  Search modes - Boolean/Phrase | Interface - EBSCOhost Research Databases  Search Screen - Advanced Search  Database - Global Heal | 43 |

Medline:

Database(s): Ovid MEDLINE ALL 1946 to June 16, 2021

Search Strategy:

| **#** | **Searches** | **Results** |
| --- | --- | --- |
| 1 | exp International Health Regulations/ | 63 |
| 2 | ("international health regulat*" or IHR or "global health securit*" or GHS).tw. | 4266 |
| 3 | 1 or 2 | 4295 |
| 4 | exp Developing Countries/ | 76884 |
| 5 | ((("developing countr*" or low) and middle income countr*) or LMIC).tw. | 20901 |
| 6 | 4 or 5 | 92034 |
| 7 | 3 and 6 | 58 |
| 8 | limit 7 to (english language and humans) | 46 |
| 9 | limit 8 to dt=20200801-20211231 | 4 |
| 10 | limit 8 to rd=20200801-20211231 | 11 |
| 11 | 9 or 10 | 11 |

Scopus:

TITLE-ABS-KEY ( *"international health regulations"*  OR  *ihr*  OR  *"global health securit*"*  OR  *ghs* )  AND  TITLE-ABS-KEY ( *"developing countr*"*  OR  *"low and middle income countr*"*  OR  *"low income countr*"*  OR  *"middle income countr*"*  OR  *lmic* )  AND  PUBYEAR  >  *2019*

**07/11/2022 search:**

**Database:**
Embase <1974 to 2022 November 04>

| **#** | **Query** | **Results from 7 Nov 2022** |
| --- | --- | --- |
| 1 | exp International Health Regulations/ | 269 |
| 2 | ("international health regulat*" or IHR or "global health securit*" or GHS).tw. | 5,915 |
| 3 | 1 or 2 | 6,080 |
| 4 | exp Developing Countries/ | 99,210 |
| 5 | ((("developing countr*" or low) and middle income countr*) or LMIC).tw. | 32,130 |
| 6 | 4 or 5 | 127,663 |
| 7 | 3 and 6 | 83 |
| 8 | limit 7 to (human and english language) | 70 |
| 9 | limit 8 to dd=20211231-20221107 | 5 |
| 10 | limit 8 to rd=20211231-20221107 | 11 |
| 11 | 9 or 10 | 16 |

Ovid Emcare <1995 to 2022 Week 44>

| **#** | **Query** | **Results from 7 Nov 2022** |
| --- | --- | --- |
| 1 | exp International Health Regulations/ | 142 |
| 2 | ("international health regulat*" or IHR or "global health securit*" or GHS).tw. | 1,655 |
| 3 | 1 or 2 | 1,708 |
| 4 | exp Developing Countries/ | 24,758 |
| 5 | ((("developing countr*" or low) and middle income countr*) or LMIC).tw. | 20,213 |
| 6 | 4 or 5 | 43,950 |
| 7 | 3 and 6 | 36 |
| 8 | limit 7 to (english language and humans) | 24 |
| 9 | limit 8 to dd=20211231-20221107 | 2 |

Ovid MEDLINE and Epub Ahead of Print, In-Process, In-Data-Review & Other Non-Indexed Citations and Daily <1946 to November 04, 2022>

| **#** | **Query** | **Results from 7 Nov 2022** |
| --- | --- | --- |
| 1 | exp International Health Regulations/ | 83 |
| 2 | ("international health regulat*" or IHR or "global health securit*" or GHS).tw. | 4,910 |
| 3 | 1 or 2 | 4,942 |
| 4 | exp Developing Countries/ | 80,000 |
| 5 | ((("developing countr*" or low) and middle income countr*) or LMIC).tw. | 27,273 |
| 6 | 4 or 5 | 99,728 |
| 7 | 3 and 6 | 68 |
| 8 | limit 7 to (english language and humans) | 52 |
| 9 | limit 8 to dt=20211231-20221107 | 4 |
| 10 | limit 8 to rd=20211231-20221107 | 9 |
| 11 | 9 or 10 | 9 |

Global Health:

| **#** | **Query** | **Limiters/Expanders** | **Last Run Via** | **Results** |
| --- | --- | --- | --- | --- |
| S3 | S1 AND S2 | Expanders - Apply equivalent subjects  Search modes - Boolean/Phrase | Interface - EBSCOhost Research Databases  Search Screen - Advanced Search  Database - Global Health | 3 |
| S2 | SU Developing Countries OR TX "developing countr*" OR TX # "low and middle income" # OR TX LMIC | Limiters - Publication Year: 20211201-20221131  Expanders - Apply equivalent subjects  Search modes - Boolean/Phrase | Interface - EBSCOhost Research Databases  Search Screen - Advanced Search  Database - Global Health | 2,131 |
| S1 | TX "international health regulat*" OR TX IHR OR TX "Global health securit*" OR TX GHS | Limiters - Publication Year: 20211201-20221131  Expanders - Apply equivalent subjects  Search modes - Boolean/Phrase | Interface - EBSCOhost Research Databases  Search Screen - Advanced Search  Database - Global Health | 54 |

Scopus

( ( TITLE-ABS-KEY ( "developing countr*" )  OR  TITLE-ABS-KEY ( "low and middle income countr*" )  OR  TITLE-ABS-KEY ( "low income countr*" )  OR  TITLE-ABS-KEY ( "middle income countr*" )  OR  TITLE-ABS-KEY ( lmic ) ) )  AND  ( ( TITLE-ABS-KEY ( "international health regulations" )  OR  TITLE-ABS-KEY ( ihr )  OR  TITLE-ABS-KEY ( "global health securit*" )  OR  TITLE-ABS-KEY ( ghs ) ) )  AND  ( LIMIT-TO ( PUBYEAR ,  2022 )  OR  LIMIT-TO ( PUBYEAR ,  2021 ) )

## References

1 Tricco, A. C. *et al.* PRISMA Extension for Scoping Reviews (PRISMA-ScR): Checklist and Explanation. *Annals of Internal Medicine* **169**, 467-473, doi:10.7326/M18-0850 (2018).

2 Preferred Reporting Items for Systematic Reviews and Meta-Analyses (PRISMA). *PRISMA for Scoping Reviews*, <<https://prisma-statement.org/Extensions/ScopingReviews>> (2022).
