# Supplementary material for: The role of international support programmes in global health security capacity building: A scoping review
Source: PLOS Glob Public Health. 2023 Apr 5;3(4):e0001763. doi: 10.1371/journal.pgph.0001763 (PMC10075474; doi:10.1371/journal.pgph.0001763)
Supplement: Attachment — Submitted filename: Doble et al - Response to Reviewers.docx [file pgph.0001763.s002.docx]

The Executive Editor & Editors-in-Chief,

PLOS Global Public Health

The Bradfield Centre, 184 Cambridge Science Park

Milton, Cambridge, CB4 0GA

United Kingdom

22^nd^ February 2023

**Re: Editors’ & reviewers’ comments on *“The role of international support programmes in global health security capacity building: an evidence review”***

Dear Editors

We thank you for your consideration and review of our manuscript entitled “The role of international support programmes in global health security capacity building: an evidence review”, and for the helpful comments.

We have addressed the Editors’ and reviewers’ comments and suggestions, and submitted a revised manuscript (both with and without tracked-changes). Full details of how we have addressed each point are outlined in the table below.

Thank you again for your comments.

Yours faithfully,

|   Dr Anne Doble  Public Health Specialty Registrar,  Halton Borough Council  Tel: +44 (0) 7790 613482  E-mail: [annedoble@hotmail.com](mailto:annedoble@hotmail.com); [anne.doble@halton.gov.uk](mailto:anne.doble@halton.gov.uk) |   Zoe Sheridan  Senior Project Officer International Health Regulations Strengthening Project,  UK Health Security Agency  Tel: +44 (0) 117 906 9010  E-mail: [zoe.sheridan@ukhsa.gov.uk](mailto:zoe.sheridan@ukhsa.gov.uk) |
| --- | --- |

**Responses to Editors’ & Reviewers’ comments:**

| **General comments** | **Authors’ Response** |
| --- | --- |
| The manuscript represents a timely and important piece. Please ensure that the topic of animal health is acknowledged in the revised submission, and that terms are defined clearly in the early sections; this will add to the conceptual framing. The reviewers have also made additional comments which will likely increase the clarity and depth of the paper, so I encourage you to consider them closely. | See responses to individual comments below |
| **Journal requirements comments** | **Authors’ Response** |
| Please identify the study as a scoping review in the title. | The study title has been amended to “The role of international support programmes in global health security capacity building: a scoping review” |
| Please send a completed 'Competing Interests' statement, including any COIs declared by your co-authors. If you have no competing interests to declare, please state "The authors have declared that no competing interests exist". Otherwise please declare all competing interests beginning with the statement "I have read the journal's policy and the authors of this manuscript have the following competing interests:" | This statement was included in the original online submission as requested: “Ebere Okereke currently serves on the editorial board of PLOS Global Public Health. The authors declare no other competing interests.” |
| Please indicate in the online submission the full and correct funding information for your study and confirm the order in which funding contributions should appear. | This information was included in the original online submission as requested: “The IHR Strengthening Project is funded by UK Official Development Assistance, through the Department of Health and Social Care GHS Programme. The funders had no role in study design, data collection and analysis, decision to publish, or preparation of the manuscript.” |
| We do not publish any copyright or trademark symbols that usually accompany proprietary names, eg (R), (C), or TM (e.g. next to drug or reagent names). Please remove all instances of trademark/copyright symbols throughout the text, including R on page 5. | All such symbols have been removed from the main manuscript & the S1 Appendix. |
| We have noticed that you have uploaded Supporting Information files, but you have not included a list of legends. Please add a full list of legends for your Supporting Information files after the references list. | This list was included originally at the end of the manuscript but before the references. It has now been moved to after the reference list. |
| Please review your reference list to ensure that it is complete and correct. If you have cited papers that have been retracted, please include the rationale for doing so in the manuscript text, or remove these references and replace them with relevant current references. Any changes to the reference list should be mentioned in the rebuttal letter that accompanies your revised manuscript. If you need to cite a retracted article, indicate the article’s retracted status in the References list and also include a citation and full reference for the retraction notice. | We have checked the accuracy and completeness of the reference list and also have run the manuscript through the Scite_ reference check. None of the cited references have been retracted. One minor change formatting change was made (addition of an end square bracket to webpage citations). In addressing the reviewers’ comments, several further citations have been added. These are listed at the end of this document. |
| **Additional Editor comments** | **Authors’ Response** |
| The manuscript presents a timely and important analysis of health security capacity strengthening and the role of international support programmes. The peer reviewers have made some pertinent suggestions for further strengthening the manuscript's arguments, as described in more detail below. I would particular recommend addressing the points raised by Reviewer #1 with respect to acknowledging animal health as a critical domain within health security, and per both reviewers, providing additional definitions in the opening sections for clarity. Please also review Reviewer #2's suggestions regarding additional references to support the description of conceptual linkages between health security, UHC, etc. The other detailed comments should also be reviewed and considered, as they will likely also provide benefit, if space allows! | Please see responses to Reviewer 1 and 2’s comments for details on how these points have been addressed. |
| **Reviewer #1 comments** | **Authors’ Response** |
| This is a well written review that adresses a topical issue in global health security. Given that most recent epidemics originate from the human-animal-enviornmental interface, I find it rather surprising that the review did not consider animal health | We have expanded on the implications of zoonotic emerging infections and the importance of collaboration between animal and human health through One Health approaches, in the ‘why’ section on partnerships. This section has also been split into two sub-headings to more clearly delineate the focus on One Health. |
| I also do feel that this review should make more pragmatic recommendations on the perspectives for the future. For example what needs to be done with respect to workforce development including having staffing norms by cadre? What are the orphan cadres that need to be trained? what curriculum review needs to be done? | See below response to similar comment from Reviewer 2 |
| On they issue of integratation of health sucurity and resilient health systems what needs to be done? For a health manager, resilience needs to be viewed from the perspective of their core focus - delivery of essential health and related services that people need. A health manager focuses on ensuring the health investments are organized and managed in a manner that maximizes access to essential services by their recipient population. Thus, resilience needs to be contextualized within the description of a functional health system for a health manager. For most health actors, a functional health system is described from the perspective of its ability to ensure access to quality essential services that are demanded by the population. Capacities needed are therefore access to services and quality of care on the supply side, with community engagement and needs for essential services on the demand side of outputs. A description of system functionality that integrates resilience has been defined as one with the ability to ensure access to quality essential services that people are demanding for, during routine and in shock events. Resilience, together with access, quality of care and community demand represent the four distinct outputs of a functional health system. Looking from this lens of a health manager seeking a functional health system, a more practical description of health systems resilience would be the ‘ability to sustain expected access to quality essential services even when threatened by shock events’. I propose that the authors offer some insights into thios view of systems resilience. | Thank you for this helpful comment. We have expanded further on this point highlighting the potential use of the WHO Building Blocks Framework to assess resilience and making clearer references to the linkages between resilience and access, quality and demand (lines 373-385). |
| **Reviewer #2 comments** | **Authors’ Response** |
| The introduction would benefit from some important context critiquing WHY current GHS/IHR efforts have failed, in order to better understand the rationale for this piece at this specific moment | This has been elaborated on within the introduction. Please see lines 43-49 |
| Background sections would also benefit from defining some core concepts, as many terms in this paper have somewhat contested definitions for different types of stakeholders (eg, "public health capacity," "health system resilience," "international support programmes") | We have now elaborated on these terms in the introduction (lines 51-54, 69-71) or subsequent sections (e.g. the discussion on health system resilience now includes a definition). |
| The intro somewhat glosses over the IHR Strengthening Project, even though this appears to be a central focus of the paper. Explaining more about this as well as how it serves as an example for other types of international support programmes may offer helpful context. | This review aimed to identify lessons and principles for both the IHR Project specifically and for other international GHS-related programmes in general. The IHR Project itself is thus not of central importance, so we did not dwell too much on it. However we have added a citation to more information on the project (line 59). |
| The noted synergy between GHS and UHC is important, offering new ways to conceptualize effective and sustainable public health capacities, but is not explained well-enough given the relative complexity and novelty of this emerging area of research. The paper could be strengthened by more comprehensively fleshing out what researchers in this space have been arguing (eg, https://www.thelancet.com/journals/lancet/article/PIIS0140-6736(23)00014-4/fulltext , https://www.thelancet.com/journals/langlo/article/PIIS2214-109X(22)00341-2/fulltext, https://www.thelancet.com/servlet/linkout?suffix=e_1_4_1_2_78_2&dbid=4&doi=10.1016/S2214-109X(22)00341-2&key=10.1016%2FS0140-6736%2821%2900029-5&cf=fulltext&site=lancet-site, https://www.uhc2030.org/news-and-stories/news/action-on-health-systems-for-universal-health-coverage-and-health-security-555531/) -- and why it is important to helping reimagine health systems in the context of health security. | We have expanded on the discussion of the synergies between GHS and UHC, emphasising the arguments for pursuing GHS and UHC strengthening in tandem (lines 346-372). |
| The structure of the paper could be improved by a brief paragraph detailing the organization and conceptual framing of these topics in the main portion (eg, did you categorize them along IHR core capacities, HSS building blocks, or something else? etc.) | We have added further detail in the Introduction to clarify the conceptual framing and the reasoning for the structure (lines 65-69). |
| While an effective review of this space, some of the potentially-most insightful information has not been fleshed out or given adequate nuance to provide a novel analysis. This could be addressed by drawing out more specific examples and unique ideas, rather than largely resorting to high-level framings. For example: "However, training or “technical skills development” based interventions often are delivered in isolation from sustainable knowledge transfer or other supportive structures, failing to recognise systemic and structural factors which cause and compound IHR-related technical capacity weaknesses [13, 17, 47-49]." -- what are the systemic and structural factors? This seems to be the most compelling finding, but is missing entirely. | The “systemic and structural factors” are those which emerged through the remaining themes which we go on to discuss through the rest of the paper (hence the mention that these are “discussed subsequently”), for instance the human resource infrastructure, sustainable funds and budgeting, or integration of efforts into wider health system strengthening. A line to clarify this has been added (line 155-156). |
| The conclusion may benefit from having more concrete recommendations (perhaps just by better organizing/structuring this section) for international practitioners to better conceptualize this work. | The Conclusion has been reworked to include a table with specific recommendations for international support programmes, relating to the themes discussed in the review. The side panel in Fig 1 (which described inputs for international support programmes) has also now been integrated into Table 1 (w have re-submitted a revised version of the figure). |

**Additional citations in revised manuscript:**

- Lal A, Abdalla SM, Chattu VK, Erondu NA, Lee T-L, Singh S, et al. Pandemic preparedness and response: exploring the role of universal health coverage within the global health security architecture. The Lancet Global Health. 2022;10(11):e1675-e83.
- UK Department for International Development. Technical Assistance and Capacity Building in International Development 2019 [Available from: https://www.gov.uk/research-for-development-outputs/technical-assistance-and-capacity-building-in-international-development.
- United Nations Academic Impact. Capacity-Building 2023 [Available from: https://www.un.org/en/academic-impact/capacity-building.
- UKHSA IHR Strengthening Project. The Global Health Network - International Health Regulations Strengthening Project page 2023 [Available from: https://gphihr.tghn.org/.
- Kishida F. Human security and universal health coverage: Japan's vision for the G7 Hiroshima Summit. The Lancet. 2023;401(10373):246-7.
- Fridell M, Edwin S, von Schreeb J, Saulnier DD. Health System Resilience: What Are We Talking About? A Scoping Review Mapping Characteristics and Keywords. Int J Health Policy Manag. 2020;9(1):6-16.
- World Health Organization. Everybody's business - strengthening health systems to improve health outcomes: WHO’s framework for action. Geneva: World Health Organization; 2007.
- Karamagi HC, Tumusiime P, Titi-Ofei R, Droti B, Kipruto H, Nabyonga-Orem J, et al. Towards universal health coverage in the WHO African Region: assessing health system functionality, incorporating lessons from COVID-19. BMJ Glob Health. 2021;6(3).
- UN Environment Programme. Preventing the next pandemic - Zoonotic diseases and how to break the chain of transmission 2020 [Available from: https://www.unep.org/resources/report/preventing-future-zoonotic-disease-outbreaks-protecting-environment-animals-and.
- Belot G, Caya F, Errecaborde KM, Traore T, Lafia B, Skrypnyk A, et al. IHR-PVS National Bridging Workshops, a tool to operationalize the collaboration between human and animal health while advancing sector-specific goals in countries. PLoS One. 2021;16(6):e0245312.
- Murphy SC, Negron ME, Pieracci EG, Deressa A, Bekele W, Regassa F, et al. One Health collaborations for zoonotic disease control in Ethiopia. Revue scientifique et technique (International Office of Epizootics). 2019;38(1):51-60.
